# Puromycin-based vectors promote a ROS-dependent recruitment of PML to nuclear inclusions enriched with HSP70 and Proteasomes

**DOI:** 10.1186/1471-2121-10-32

**Published:** 2009-05-01

**Authors:** Diarmuid M Moran, Hong Shen, Carl G Maki

**Affiliations:** 1 Department of Radiation and Cellular Oncology, University of Chicago, Chicago, Illinois, USA

## Abstract

**Background:**

Promyelocytic Leukemia (PML) protein can interact with a multitude of cellular factors and has been implicated in the regulation of various processes, including protein sequestration, cell cycle regulation and DNA damage responses. Previous studies reported that misfolded proteins or proteins containing polyglutamine tracts form aggregates with PML, chaperones, and components of the proteasome, supporting a role for PML in misfolded protein degradation.

**Results:**

In the current study, we have identified a reactive oxygen species (ROS) dependent aggregation of PML, small ubiquitin-like modifier 1 (SUMO-1), heat shock protein 70 (HSP70) and 20S proteasomes in human cell lines that have been transiently transfected with vectors expressing the puromycin resistance gene, puromycin n-acetyl transferase (pac). Immunofluorescent studies demonstrated that PML, SUMO-1, HSP70 and 20S proteasomes aggregated to form nuclear inclusions in multiple cell lines transfected with vectors expressing puromycin (puro) resistance in regions distinct from nucleoli. This effect does not occur in cells transfected with identical vectors expressing other antibiotic resistance genes or with vectors from which the pac sequence has been deleted. Furthermore, ROS scavengers were shown to ablate the effect of puro vectors on protein aggregation in transfected cells demonstrating a dependency of this effect on the redox state of transfected cells.

**Conclusion:**

Taken together we propose that puromycin vectors may elicit an unexpected misfolded protein response, associated with the formation of nuclear aggresome like structures in human cell lines. This effect has broad implications for cellular behavior and experimental design.

## Background

The human promyelocytic leukemia PML gene was initially identified as the fusion partner of the retinoic acid receptor α gene in the T(15;17) chromosomal translocation in acute promyelocytic leukemia [1]. The PML protein localizes to punctate nuclear structures called PML nuclear bodies (NBs) which are discrete foci (0.1–0.2 μm diameter) that are present in most mammalian cells. PML is essential for the formation of PML NBs, however the bodies themselves are composed of many other proteins such as SUMO, SP100, CBP and Daxx. SUMO-1 (Small Ubiquitin like Modifier 1) belongs to a family of ubiquitin like proteins that may be covalently attached to protein substrates and are typically associated with protein localization. SUMO modification of PML is required for NB formation [1,2]. Thus far over 50 proteins have been identified to reside, either permanently or more typically transiently, in PML NBs. Typically the size, localization and number of PML NBs is well conserved in most cells, however they are dynamic structures that change size, position and number in response to stress such as heat shock, DNA damage and heavy metal exposure [2]. PML NBs have been implicated in the regulation of many cellular functions including DNA repair, antiviral responses, apoptosis, proteasomal degradation, senescence and gene regulation. It is still unclear how PML carries out its functions but it is recognized that the localization of proteins to NBs is important [3]. It has been suggested that PML NBs may operate as storage sites for nuclear proteins and/or act as catalytic surfaces where proteins accumulate to be post translationally modified [4,5]. It has also been suggested that PML NBs may act as active sites for specific nuclear functions such as chromatin regulation and transcription [6,7]. PML is nonessential and PML knockout mice are viable, but the protein has been linked to many pathological states [8]. In particular, PML has been characterized as a tumor suppressor due to its functions in apoptosis, genomic stability and senescence [3]. PML NBs also play a role in neurodegenerative diseases however its participation is not very well defined. Nevertheless, PML NBs are often localized in the nuclear inclusions formed by protein/polyglutamine aggregates in diseases such as spinocerebrellar ataxia and Huntington's disease [9,10].

Newly synthesized proteins must fold correctly to become functional molecules. Many events including errors in transcription, mRNA processing and translation lead to failure in achieving correct protein conformation [11]. Furthermore, environmental factors such as oxidative, thermal or osmotic stress can interfere with folding of nascent polypeptides and can damage correctly folded proteins or intermediates [12,13]. Misfolded proteins expose hydrophobic residues that are normally buried in the protein's interior leading to protein aggregation. Cells have evolved 'quality control' systems to minimize protein misfolding and prevent protein aggregation. Molecular chaperones such as the Heat Shock Proteins (HSP) bind to and stabilize exposed hydrophobic residues thereby reducing the likelihood of protein aggregation [14]. Chaperones such as HSP70 are also believed to assist in refolding misfolded proteins. In addition, misfolded proteins are generally targeted for degradation by the ubiquitin proteasome system [13]. Proteasomes are multi subunit complexes that are abundant in the nucleus and cytosol in all eukaryotic cells that function to degrade unwanted or damaged proteins. Typically proteins are covalently modified with one or more ubiquitin molecules that act as a tag for recognition by the proteasomal complex. Failure of these quality control systems leads to protein aggregates which are difficult to unfold and/or degrade and are associated with the etiologies of many degenerative diseases such as amyloidoses, Alzheimer's disease and Parkinson's disease [14,15].

In the current study we have identified a reactive oxygen species (ROS) dependent aggregation of proteins (including PML), proteasomes and chaperones into nuclear inclusions in cells lines that have been transfected with vectors expressing the puromycin resistance gene. This response is strikingly similar to previously described misfolded protein responses and has broad implications for cellular behavior and experimental design.

## Results

**Figure 1:**
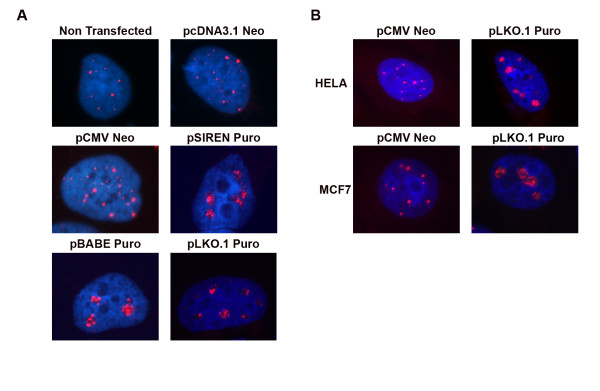
**Transfection with pSIREN Puro, pBABE Puro or pLKO.1 Puro vectors causes PML aggregation in multiple cell types**. (A) U2OS cells on glass coverslips were transiently transfected with empty vectors using Fugene transfection reagent and immunostained for PML (red) 48 h after transfection. PML antibodies were detected using a rhodamine-x conjugated goat anti mouse IgG. Cell nuclei were counterstained with DAPI (blue). Images were captured at 100× magnification. (B) MCF-7 and HELA cells on glass coverslips were transiently transfected with pCMV Neo or pLKO.1 PURO empty vector using Fugene transfection reagent and immunostained for PML (red) 48 h after transfection. PML antibodies were detected using a rhodamine-x conjugated goat anti mouse IgG. Cell nuclei were counterstained with DAPI (blue). Images were captured at 100× magnification.

During the course of our studies we observed an apparent relocalization of PML nuclear bodies (NBs) in cell lines transfected with shRNA vectors targeting specific E3 ligases (MDM2 and CHIP). Surprisingly, we also observed this effect in cells transfected with control vectors expressing non targeting shRNA sequences and those without DNA inserts (empty vectors). To examine this further, U2OS cells were transiently transfected with empty vectors using Fugene transfection reagent and immunostained for PML 24 h and 48 h after transfection. PML localized to discrete foci (11 ± 5 foci per cell) dispersed throughout the nuclei of non transfected and mock transfected cells (Fig 1A). These foci had the typical size and appearance of PML NBs [3]. Transfection with pcDNA3.1 Neo or pCMV Neo empty vectors had no apparent effect on PML localization (Fig 1A). However, cells transfected with pBABE Puro, pSIREN Puro and pLKO.1 Puro empty vectors displayed aggregation of PML at multiple nuclear sites. This aggregation appeared as clustering and/or clumping of PML, and was apparent 24 h and 48 h after transfection. To test the generality of this effect, we transfected MCF7 and HeLa cells with these empty vectors. pCMV Neo and pcDNA3.1 empty vectors had no effect on PML localization in these cells (data not shown). However, transfection with pBABE Puro, pSIREN Puro and pLKO.1 Puro (Fig 1B) resulted in aggregation of PML in the nuclei of both MCF7 and HeLa cells.

**Figure 2:**
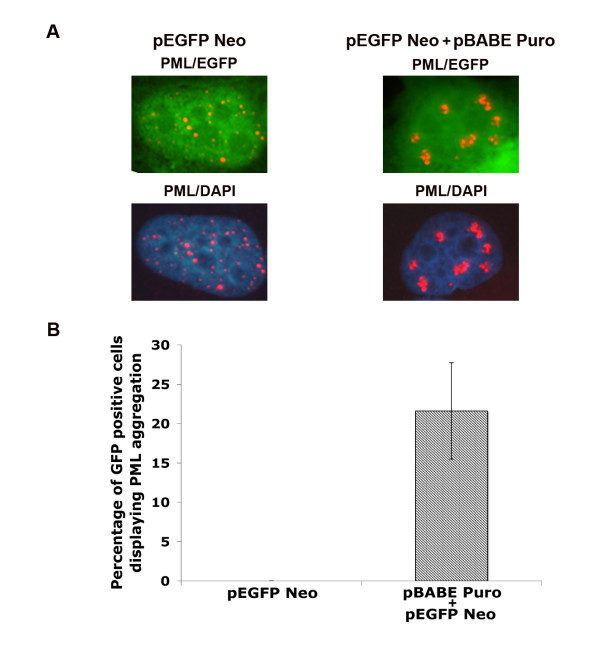
**PML aggregation occurs specifically in transfected cells**. pEGFP Neo empty vector was transiently cotransfected with pBABE Puro empty vector in U2OS cells on glass coverslips. Cells were immunostained for PML (red) 48 h after transfection. PML antibodies were detected using a rhodamine-x conjugated goat anti mouse IgG. Cell nuclei were counterstained with DAPI (blue). Transfected cells were identified by EGFP expression (green). (A) Representative images of U2OS cells transfected with pEGFP Neo empty vector alone or pEGFP/pBABE Puro demonstrating PML aggregation in co-transfected cells. Images captured at 100× magnification. (B) Percentage of co-tansfected EGFP positive cells displaying PML aggregation were quantified. Percentage represents average ± SEM of five independent experiments with 300 cells counted per experiment.

We next aimed to quantify the percentage of transfected cells in which PML aggregation occurred. To this end, pEGFP Neo empty vector was cotransfected with pBABE Puro into U2OS cells, and the cells immunostained for PML 48 h after transfection. Transfected cells were identified by EGFP expression. PML localization was not altered in cells transfected with pEGFP Neo alone (Fig. 2A). In contrast, pBABE Puro (Fig 2A) empty vectors caused PML aggregation in EGFP co-expressing cells. Transfection of pBABE Puro caused PML NB aggregation in 21 ± 6% of EGFP expressing cells (Fig 2B).

**Figure 3:**
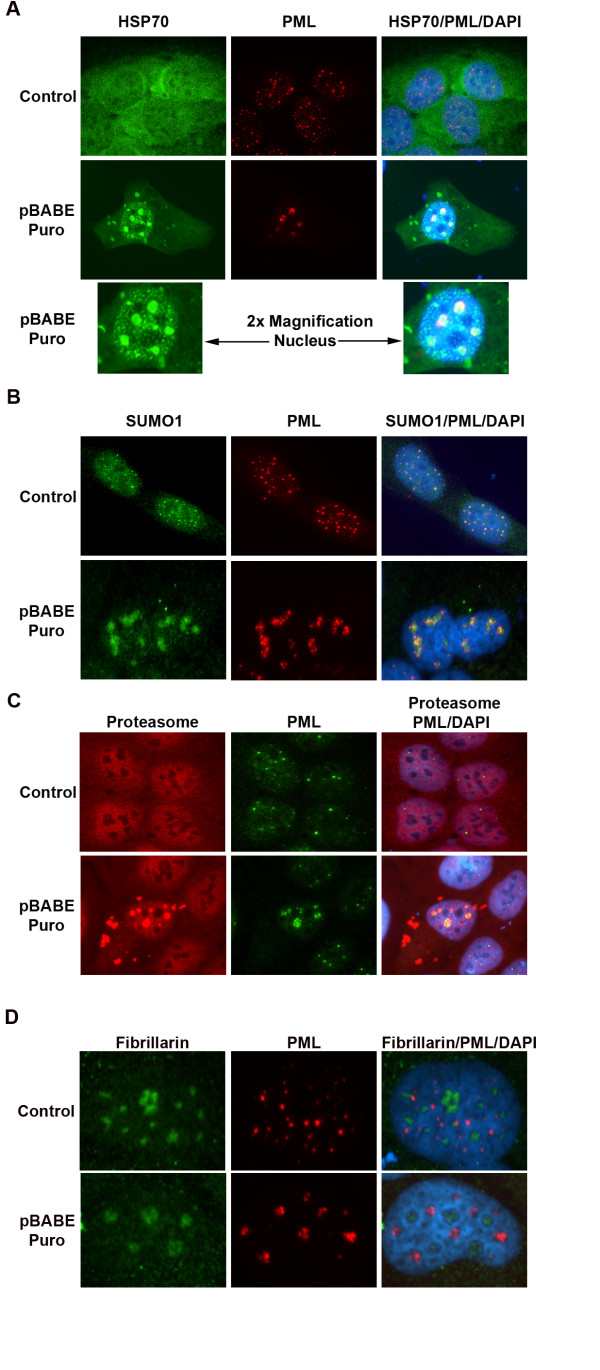
**PML aggregates colocalize with HSP70, SUMO-1 and 20S proteasomes in regions distinct from nucleoli in pBABE Puro transfected cells**. U2OS cells on glass coverslips were transiently transfected with pCMV Neo (Control) or pBABE Puro empty vector and immunostained 48 h after transfection. Cell nuclei were counterstained with DAPI (blue). Images were captured at 63× (A-C) or 100× (D) magnification. (A) Representative images of cells immunostained for HSP70 (green) and PML (red). PML and HSP70 antibodies were detected using a rhodamine-x conjugated goat anti mouse IgG and Alexa 488 goat anti rabbit IgG respectively. Lower panel displays magnified nuclei (2×) from pBABE Puro transfected cells (panel above) in order to display speckled nuclear staining pattern of HSP70 in transfected cells. (B) Representative images of cells immunostained for SUMO-1 (green) and PML (red). PML and SUMO-1 antibodies were detected using a rhodamine-x conjugated goat anti mouse IgG and Alexa 488 goat anti rabbit IgG respectively. (C) Representative images of cells immunostained for 20S Proteasomes (red) and PML (green). 20S Proteasomes and PML antibodies were detected using a rhodamine-x conjugated goat anti mouse IgG and Alexa 488 goat anti rabbit IgG respectively. (D) Representative images of cells immunostained for Fibrillarin (green) and PML (red). PML and HSP70 antibodies were detected using a rhodamine-x conjugated goat anti mouse IgG and Alexa 488 goat anti rabbit IgG respectively.

PML aggregates similar to that observed in Figs 1 and 2 have been described previously in association with misfolded proteins that accumulate in response to proteasome inhibition, viral protein expression, and in polyglutamine diseases such as Huntington's and spinocerebrellar ataxias [9,10,16,17]. PML was found coaggregated in these nuclear inclusions with SUMO-1, HSP chaperones, and proteasomes. We examined SUMO-1, HSP70, and proteasome localization in cells transfected with the empty, puromycin-resistance vectors. U2OS cells were transiently transfected with pBABE Puro or pCMV Neo (control) empty vectors and immunostained 48 h later for PML and either HSP70, SUMO-1 or 20S proteasomes. HSP70 was detected diffusely in both the nucleus and cytoplasm of non-transfected and control U2OS cells (Fig 3A). In pBABE Puro transfected cells, HSP70 was dramatically relocalized to both nuclear and cytoplasmic aggregates. Large nuclear aggregates of HSP70 colocalized with PML clusters in all cells examined. Smaller, speckled HSP70 aggregates were also observed throughout the nuclei of many transfected cells but these usually did not colocalize with PML aggregates (Fig 3A, lower panel/magnified nuclei). SUMO-1 was detected in distinct nuclear foci that colocalized with PML in non-transfected and control cells (Fig 3B). In pBABE Puro transfected cells SUMO-1 relocalized to regions of PML aggregation, either colocalized with aggregated PML or in the area immediately surrounding PML aggregates. Immunostaining using antibodies against 20S proteasomes demonstrated diffuse nuclear and cytoplasmic localization in non transfected and control cells (Fig 3C). In pBABE Puro transfected cells, 20S proteasomes were relocalized to large cytoplasmic and nuclear aggregates which colocalized with nuclear PML aggregates (Fig 3C) and HSP70 aggregates in both the nucleus and cytoplasm (data not shown). Relocalization of HSP70, SUMO-1, and 20S proteasomes was also observed in pLKO.1 Puro and pSUPER Puro transfected U2OS cells (data not shown). In addition, these vectors caused a similar relocalization of HSP70, SUMO-1, and 20S proteasomes in MCF-7 and HELA cells (data not shown). Nucleolar localization of PML has been described during proteasomal inhibition and thermal stress [17]. To examine the sub-nuclear localization of PML, we immunostained pBABE Puro transfected cells for both PML and Fibrillarin as a nucleolar marker. PML did not colocalize with Fibrillarin stained regions (nucleoli) in non transfected or control cells (Fig 3D), and PML aggregates also did not colocalize with Fibrillarin in pBABE Puro transfected cells. This indicates the PML aggregates are not localized in nucleolar regions. In summary, transfection of pBABE Puro, pSUPER Puro and pLKO.1 Puro caused aggregation of PML, HSP70, SUMO-1 and 20S Proteasomes in nuclear regions distinct from nucleoli.

**Figure 4:**
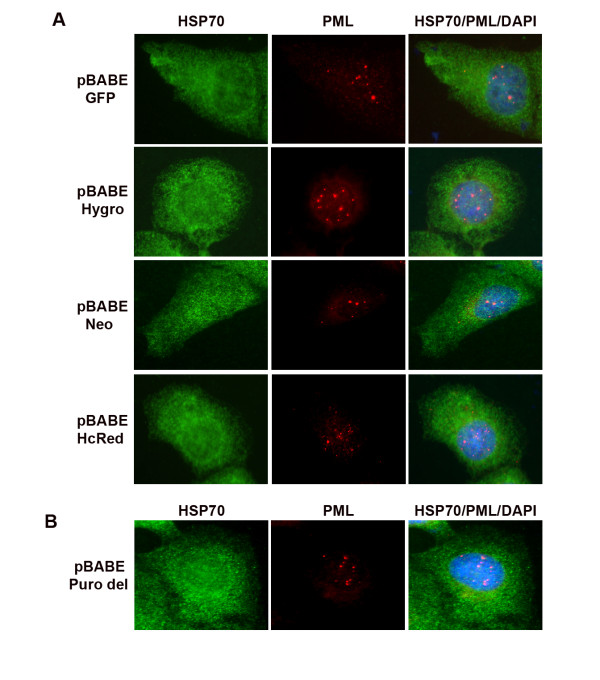
**PML/HSP70 co-aggregation is dependent upon the presence of the pac resistance gene sequence in puromycin vectors**. U2OS cells on glass coverslips were transiently transfected with empty vectors and immunostained for HSP70 (green) and PML (red) 48 h after transfection. PML antibodies were detected using a rhodamine-x conjugated goat anti mouse IgG or Cy5 goat anti mouse IgG/pseudocolored red (pBABE hcRed transfected cells only). HSP70 antibodies were detected using Alexa 488 goat anti rabbit IgG or Cy5 goat anti rabbit IgG/pseudocolored green (pBABE GFP transfected cells only). Cell nuclei were counterstained with DAPI (blue). Images were captured at 100× magnification. (A) Representative images of cells transfected with multiple pBABE vectors carrying alternative selection markers demonstrating absence of PML/HSP70 aggregation. (B) Representative images of cells transfected with pBABE Puro del demonstrating absence of PML/HSP70 aggregation. pBABE Puro del was generated by removal of the pac gene sequence from parental vector pBABE PURO.

Next, we wished to address why this effect was specific to certain vectors but not others. The empty vectors that caused PML, HSP70, SUMO-1, and 20S proteasome aggregation (pBABE Puro, pLKO.1 Puro and pSUPER Puro) are retroviral vectors that express the puromycin resistance gene. The vectors that did not cause this effect (pcDNA3.1 Neo, CMV Neo and pEGFP Neo) are non-retroviral vectors that express the neomycin resistance gene. We questioned whether this effect could be related to either retroviral genetic sequences or expression of the puromycin resistance gene, puromycin n-acetyl transferase (pac). In order to investigate this, we used multiple pBABE vectors which differed only in the selection marker expressed (Puro, Hygro, Neo, GFP, HcRed). U2OS cells were transfected with these pBABE vectors and immunostained 48 h later for HSP70 and PML. While pBABE Puro caused PML and HSP70 coaggregation in transfected cells (as shown in Figs 1, 2, 3), none of the other pBABE vectors had this effect (Fig 4A). This suggested that the puromycin resistance gene was responsible for PML and HSP70 aggregation. Next, we deleted the pac gene sequence from the pBABE Puro vector and transfected the resultant vector (named pBABE Puro del) into U2OS cells. Immunostaining for PML and HSP70 demonstrated that neither PML nor HSP70 were aggregated in cells transfected with pBABE Puro del (Fig 4B). Collectively, this data demonstrates the pac gene is required for PML and HSP70 coaggregation.

**Figure 5:**
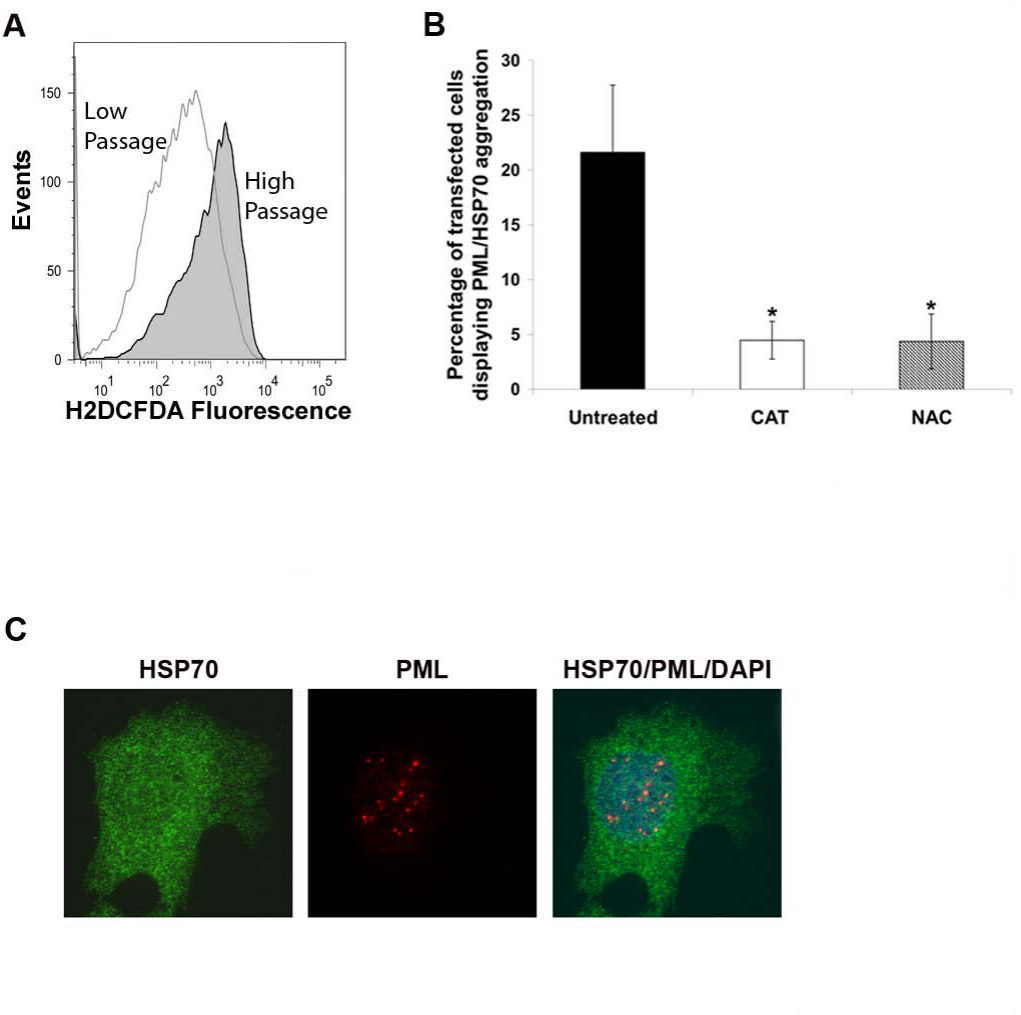
**PML/HSP70 co-aggregation is dependent upon ROS and is not maintained in puromycin resistant selected cells**. (A) Low passage (<10 passages) and high passage U2OS cells were incubated with H2DCFDA and analyzed by flow cytometry to detect ROS levels. Representative flow cytometry histogram displaying levels of fluorescent H2DCFDA in low passage versus high passage U2OS cells. (B) U2OS cells on glass coverslips were transiently transfected with pBABE Puro or pEGFP Neo. ROS scavengers, catalase (CAT; 50 μg/mL) or N acetyl cysteine (NAC; 10 mM), were added to media of transfected cells 15 h after transfection. Transfected cells were immunostained for HSP70 and PML 48 h after transfection. Cells displaying aggregation of PML/HSP70 were quantified and expressed as a percentage of EGFP expressing cells. Data represents the average ± SEM from 3 independent experiments. Asterisk represents p-value < 0.01 compared to cells without ROS scavengers (Student T Test). (C) Representative images of puromycin selected cells demonstrating the absence of PML/HSP70 aggregation. U2OS cells were transfected with pBABE puro and selected in puromycin as described in materials and methods and then immunostained for HSP70 (green) and PML (red). PML and HSP70 antibodies were detected using a rhodamine-x conjugated goat anti mouse IgG and Alexa 488 goat anti rabbit IgG respectively. Cell nuclei were counterstained with DAPI (blue). Images were captured at 100× magnification.

In the course of these experiments, we observed that high-passage U2OS cells were prone to PML aggregation following transfection with Puro-based vectors. However, when these experiments were repeated in low passage U2OS cells (<10 passages) we did not detect any aggregation (n = 3 separate experiments). Cell lines are often maintained in culture over long periods and are subjected to frequent passaging and multiple freeze-thaw cycles. Notably, ROS levels have been reported to increase in cells maintained in culture for prolonged periods, and multiple studies have linked elevated ROS levels with protein aggregation and misfolding [13,18,19]. We hypothesized that oxidative stress/ROS generation in cells maintained in culture over long periods might influence PML aggregation in transfected cells. To investigate this, we first compared the level of ROS in high passage and low passage U2OS cells using a flow cytometry approach based on H2DCFDA, a cell-permeant indicator for reactive oxygen species that fluoresces in a quantifiable manner in response to oxidation levels within the cell. Flow analysis to detect fluorescent H2DCFDA demonstrated that high passage U2OS cells contain 2.7 ± 0.69 (n = 3 separate experiments) fold higher levels of ROS compared to low passage cells (Fig 5A). Next we investigated whether these higher levels of ROS may be responsible for the protein aggregation observed in high passage cells. To this end, aggregation-prone (high passage) U2OS cells were transfected with pBABE Puro or pEGFP Neo. ROS scavengers, N-acetyl cysteine (NAC) or catalase, were then added to the media of transfected cells (15 h later), and the cells immunostained 48 h after transfection for PML and HSP70. EGFP expressing cells were quantified as a measure of tranfection efficiency under each condition. Cells displaying aggregation of PML/HSP70 were quantified and values corrected for transfection efficiency. PML/HSP70 aggregation in NAC and catalase treated cells was reduced by 80% (Fig 5B). This data suggests that ROS levels enhance PML and HSP70 aggregation in these cells.

Finally, we examined whether transfected cells maintain protein aggregation after undergoing puromycin selection. For this, U2OS cells were transfected with pBABE puro or left non-transfected. Four days later, cells were placed in media containing puromycin (0–15 μg/mL). Non-transfected cells were killed at all doses of puromcyin 2 days after treatment. Transfected cells were grown for two weeks in puromycin in order to select for stable, puro-resistant cells. Stable cells were seeded on coverslips in the presence or absence of puromycin and stained 3 days later for PML and HSP70. Analysis of transfected cells grown in the absence of puromycin demonstrated these cells do not display PML and/or HSP70 aggregation (Fig 5C). Similarly, stable resistant cells grown in the presence or absence of puromycin (3 days), no longer display aggregation of PML and/or HSP70.

## Discussion

In the current study, we have described a ROS sensitive aggregation of PML, HSP70, SUMO-1 and 20S proteasomes into nuclear inclusions in cells transfected with vectors expressing the selection marker gene, puromycin n-acetyl transferase (pac). Moreover, aggregation of HSP70 and proteasomes was also observed in the cytoplasm of transfected cells. Cytoplasmic inclusions containing HSP70 and proteasomes have been previously described and are associated with aggregates of misfolded proteins [20-22]. Misfolded proteins generated by either protein over-expression, mutation, or cellular stress expose hydrophobic residues leading to non productive cellular aggregates throughout the cell [11]. In the cytoplasm it has been proposed that these aggregates are transported to the microtubule organizing center (MTOC) in a microtubule dependent manner where they coalesce to form aggresomes [11,22]. Aggresomes are typically enriched with chaperones such as HSP70 and proteasomes, and are thought to be sites of misfolded protein degradation. Similarly, nuclear inclusions enriched for chaperones and proteasomes have been described in cells expressing proteins that contain expanded polyglutamine (polyQ) repeats [20,23]. In neurodegenerative disorders, such as Huntington's disease and spinocerebellar ataxias, mutant proteins (huntingtin and ataxins respectively) containing expanded polyQ repeats form nuclear inclusions associated with proteasomes and HSP70 in neuronal cells [9,20,23]. Notably, many reports have described the presence of PML and SUMO-1 in these neurodegenerative nuclear inclusions [9,10,24]. Moreover, nuclear inclusions containing HSP70, proteasomes and PML have been well characterized in cells overexpressing non-PolyQ aggregation prone proteins, viral proteins and in cells treated with proteasomal inhibitors [16,17,25-27]. Collectively, these studies suggest that PML bodies can represent nuclear aggresomes and potential sites of nuclear misfolded protein degradation. Based on these previous studies, we propose that a protein aggregation/misfolded protein response may account for nuclear aggregation of PML, HSP70, SUMO1 and proteasomes into nuclear inclusions in this study. We observe nuclear and cytoplasmic aggregates in cells transfected with puro vectors which are strikingly similar to cellular aggresomes. Previous studies have shown aggregation of transiently overexpressed proteins, however this has not been previously described in cells transfected with empty vectors [22,25]. Cells transfected with a puro vector and then selected in puromycin did not display PML/HSP70/proteasome/SUMO aggregates. This suggests cells with aggregates may either die, or clear/resolve the aggregates through autophagy or other mechansisms [27,28].

Our results demonstrate that protein aggregation in cells transfected with empty puro vectors depends on the presence of the puromycin-resistance (pac) gene. It is presently unclear why this effect is specific to pac and why similar aggregation does not occur upon transfection with vectors expressing other selection markers. One possible reason could be that puromycin n-acetyl transferase protein (PAC) is more aggregation prone than other antibiotic resistance proteins. We propose that PAC protein may colocalize with both nuclear and cytosolic aggregates and that the presence of proteasomes and chaperones may represent the cells response to aggregated PAC protein. However, in the absence of commercially available antibodies specific to PAC, we were unable to confirm this hypothesis. The composition and the primary structure of a polypeptide determine to a large extent its propensity to aggregate. We have used two recently developed bioinformatics software programs as a preliminary approach to compare the protein sequences of neomycin, puromycin and hygromycin resistance gene products for aggregation propensity (data not shown). The software Aggrescan predicts aggregation hotspots in protein sequences [29]. Using this software we did not detect any apparent differences in aggregation propensity based on protein sequence/amino acid content. However, using a second piece of software, PONDR VL-XT (Molecular Kinetics, Inc., IN) we have detected that a greater percentage of the puromycin resistance protein (38%) is naturally disordered compared to hygromycin (25%) and neomycin (22%) resistance proteins [30]. Disordered regions (DRs) are entire proteins or regions of proteins which lack a fixed tertiary structure, essentially being partially or fully unfolded which have been linked to aggregation propensity [31]. It is tempting to speculate that the percentage of disorder in PAC may lead to its aggregation; however, it is presently unclear whether this could contribute to the observed phenotype. Other potential reasons for aggregation may include possibly higher expression levels, a greater sensitivity to ROS damage and/or a higher rate of degradation for PAC which have been shown to contribute to aggregation propensity in other studies [19,32]. It also cannot be ruled out that PAC activity or its interaction with other native proteins may contribute to protein aggregation in transfected cells. The specificity of this effect to pac is of great interest and warrants further study.

During the course of this study, we observed the effect of puro vectors on protein aggregation in multiple cell types but not in low passage/newer cells (<10 passages). Many studies have described the generation of reactive oxygen species (ROS) in cultured cells and higher levels of ROS are associated with protein aggregation [13,18,19,33]. We proposed that older cells may contain higher levels of ROS which could account for aggregation being limited to higher passage cells. Initially we identified that high passage cells have a higher level of intracellular ROS. To investigate this hypothesis further, we used two scavengers that reduce ROS through different mechanisms. Catalase functions to catalyze the decomposition of H_2_O_2_, the major cellular source of ROS, to water and oxygen. H_2_O_2 _readily passes through cell membranes allowing extracellular catalase (in cell medium) to drain H_2_O_2 _from cells thereby protecting against extracellular and intracellular H_2_O_2 _[18]. N-acetyl cysteine functions to stimulate expression of endogenous intracellular ROS scavengers, such as glutathione peroxidase [34]. Both ROS scavengers blocked protein aggregation in aggregation prone U2OS cells in this study. Since these scavengers use different mechanisms to decrease ROS, we consider this strong evidence that ROS contributes to aggregation. ROS are typically generated as the byproduct of cellular metabolic processes and are carefully controlled by cellular antioxidants or scavengers. However, oxidative stress ensues when an imbalance occurs between ROS generation and antioxidant defenses in favor of the former. Excessive ROS leads to oxidation of cellular proteins rendering them less active, less thermostable and more hydrophobic [13]. It is possible that overexpressed PAC protein is directly damaged by ROS in these cells, leading to increased hydrophobicity and aggregation. Most oxidative damage cannot be reversed and oxidized proteins are generally removed by proteasomal degradation. Proteasomal activity is also impaired by ROS and such inhibition of proteasomes may also contribute to aggregation in our model [35,36]. Many aspects of cell culture could account for excessive ROS generation in these cells. Cell culture media tends to be pro-oxidant due to components such as ascorbate, flavonoids and thiols which undergo rapid oxidation to generate H_2_O_2 _and other ROS [37-39]. Cell media is also often deficient in many antioxidants and antioxidant precursors [40,41]. Furthermore, cultured cells are grown at high oxygen tension levels (15–100 times greater than in vivo) which can lead to enhanced ROS generation by increased leakage of electrons from electron transport chains [18]. Finally, an association between ROS generation and the frequency of freeze-thaw cycles of cells has been identified [42]. We suggest that continuous culture of cell lines may render cells more susceptible to oxidative stress and that this may alter cell behavior.

## Conclusion

This study has broad implications for cell experimentation. Many studies are based on malignant cell lines that are often maintained in culture and/or frozen over long periods. Cell lines are generally considered to be more robust than primary cells which often undergo stress during cell culture; however, our study shows that cell lines can also be susceptible to oxidative stress during prolonged culture. Our study clearly shows an alteration in cell phenotype/behavior in a ROS dependent manner. The aggregation of critical multifunctional proteins and complexes such as HSP70, PML and proteasomes into nuclear inclusions clearly may affect multiple cellular processes and may lead to inaccurate conclusions in experimental studies. Cell transfection itself is a critical component of cell biology studies. Typically DNA vectors are used without consideration for the effects of resistance proteins in transfected cells. It is surprising that the puromycin resistance gene can cause these effects in cells however it is worth noting that this gene expresses a foreign protein in mammalian cells. We suggest caution when using puromycin-based DNA vectors, as these vectors may elicit an unexpected misfolded-protein response that includes PML, HSP70, SUMO-1, and proteasome coaggregation, and that could alter the outcome or interpretation of experiments.

## Methods

### Cells, Reagents and Plasmids

HeLa, MCF-7 and U2OS cells were obtained from ATCC and were grown in Dulbecco's modified Eagle's medium (DMEM) supplemented with 10% fetal bovine serum (FBS), penicillin (100 U/mL) and streptomycin (100 μg/mL). PBABE Puro, pBABE Hygro, pBABE Neo, pBABE GFP, pBABE hcRed and pSUPER plasmids were obtained from Addgene (Cambridge, MA). PLKO.1 Puro plasmid, N-Acetyl Cysteine, Bovine Catalase and puromycin were obtained from Sigma Aldrich (St Louis, MO). Fugene-6 transfection reagent was obtained from Roche (Nutley NJ).

### Transient and Stable Transfection

Fugene transfection reagent was equilibrated in serum free DMEM prior to mixing with vector DNA (2 μg). Fugene/DNA was incubated at room temperature prior to being added dropwise to cells. To establish stable cell lines, media was refreshed on transfected cells 4 days later with media containing puromycin (0, 1, 5, 10 and 15 μg/mL). Transfected cells were treated with puromycin, refreshed every 3 days, for > 2 weeks.

### Immunofluorescence

Immunofluorescent staining was performed as previously described [43]. Briefly, cells were transfected on glass coverslips in 6 well dishes. Cells were fixed in 4% paraformaldehyde and then methanol fixed in ice cold methanol. Coverslips were next blocked and permeabilized in 1% bovine serum albumin (BSA), 0.1% Triton X-100 in PBS and then incubated with primary antibodies (1:100 dilution in PBS/1% BSA for all antibodies except 1:50 dilution for HSP70) for 1.5 h. Cells were incubated with rhodamine conjugated goat anti-mouse IgG, Cy-5 conjugated goat anti rabbit or mouse IgG (1:2000 dilution in PBS/1%BSA; Jackson Immunoresearch, West Grove, PA) and/or Alexa 488 conjugated goat anti rabbit or mouse IgG (Invitrogen, Eugene, OR) for 1 h with DAPI. Primary antibodies used were specific against PML (PGM3), PML (H238), SUMO-1 (FL-101) and Fibrillarin (A-16) (Santa Cruz Biotechnology, Santa Cruz, CA); HSP70 (D69) (Cell Signaling Technology, Danvers, MA) and 20S proteasome mAb (Biomol International, UK). Fluorescent staining was visualized using an axiovert 200 fluorescence microscope (Zeiss, Göttingen, Germany).

### Deletion of pac sequence from pBABE Puro

Primers were designed for flanking regions of the pac sequence in the pBABE Puro plasmid. Deletion of the pac gene was performed using the Quikchange XL mutagenesis kit (Strgatagene, La Jolla, CA) exactly as the manufacturer described and the resulting plasmid was renamed pBABE Puro del. Deletion of the pac sequence was confirmed by DNA sequencing of the pBABE Puro del plasmid.

### Detection of intracellular ROS

Cells were grown to 70% confluency in 35 mm dishes. Cells were trypsinized and incubated with 25 μM Carboxy H_2_DCFDA (Invitrogen, Carlsbad, CA) for 30 min at 37°C in dark. Cells were next washed with ice-cold PBS. Fluorescence was quantified by flow cytometry using a BD FACSCanto (BD, Franklin Lakes, NJ). Data was analyzed using FlowJo 8.8. Geometric Mean fluorescence was used as average fluorescence value for each population.

## Authors' contributions

DM conceived, designed and performed the experimental work for this study and wrote this manuscript. HS performed the ROS detection experiments (Figure 5A). CGM participated in the design and coordination of the study.
